# Beeinflusst NoL-Monitoring den Opioidbedarf bei Da-Vinci-Prostatektomien?

**DOI:** 10.1007/s00101-022-01126-7

**Published:** 2022-05-25

**Authors:** F. Niebhagen, C. Golde, T. Koch, M. Hübler

**Affiliations:** 1 Klinik und Poliklinik für Anästhesiologie und Intensivtherapie, Universitätsklinikum Carl Gustav Carus, TU Dresden, Fetscherstr. 74, 01307 Dresden, Deutschland; 2 Klinik für Anästhesiologie, Intensivmedizin und Schmerztherapie, Krankenhaus St. Joseph-Stift Dresden (Lehrkrankenhaus der TU Dresden), Dresden, Deutschland

**Keywords:** Nozizeption, Sufentanil, PMD200, Schmerzmonitor, Allgemeinanästhesie, Pain, Nociception, Analgesics, Multiparametric, General anesthesia

## Abstract

**Hintergrund:**

Die Gabe von Opioiden zur Schmerzunterdrückung spielt eine zentrale Rolle in der modernen Anästhesiologie. Messungen von Hypnosetiefe und Muskelrelaxierung sind im Gegensatz zur Schmerzmessung seit Jahren etabliert. Seit Kurzem ist das PMD200 („Pain Monitoring System“; Fa. Medasense Biometrics™ Ltd., Ramat-Gan, Israel) verfügbar. Dieser Schmerzmonitor misst nichtinvasiv und errechnet einen dimensionslosen Schmerzindex („nociceptor level“, NoL). Die Validität und Zuverlässigkeit des Verfahrens sind Gegenstand von klinischen Studien.

**Fragestellung:**

Reduziert die Verwendung des PMD200 die Gabe von Analgetika während einer Da-Vinci-Prostatektomie?

**Material und Methoden:**

In die Studie wurden 50 Patienten aufgenommen. Nach gewichtsadaptierter Sufentanilgabe zur Narkoseinduktion und einem 10 µg Bolus vor Hautschnitt erfolgte die intraoperative Analgesie durch subjektive Entscheidung (CONT) oder aufgrund eines erhöhten NoL-Index (INT). Die statistische Auswertung erfolgte durch Mann-Whitney-U-, Kolmogorow-Smirnow-Test und Levene-Statistik.

**Ergebnisse:**

In der INT-Gruppe war die Anzahl der Sufentanilboli/h nicht signifikant geringer als in der CONT-Gruppe (*p* = 0,065). Die Varianz der Sufentanilgaben unterschied sich signifikant (*p* = 0,033). In der CONT-Gruppe war die Applikation normal verteilt (*p* = 0,2), in der INT-Gruppe hingegen nicht (*p* = 0,003).

**Diskussion:**

Eine mögliche Interpretation der Daten ist, dass die Schmerzmittelgabe in der INT-Gruppe individualisierter erfolgte, d. h., es wurden nichterforderliche Schmerzmittelgaben vermieden, und gleichzeitig detektierte das NoL-Monitoring einzelne Patienten mit deutlich erhöhtem Schmerzmittelbedarf. Diese Schlussfolgerung ist nur unter der Voraussetzung zulässig, dass das PMD200 auch tatsächlich die Entität Schmerz misst.

## Abstract (en)

**Background:**

Administration of opioids to suppress pain plays a major role in modern anesthesia. Measuring depth of hypnosis and neuromuscular recovery are already well established, and devices for pain monitoring are available. Nonetheless pain monitoring is rare in clinical practice. Recently, the pain monitoring device PMD200 (Medasense Biometrics™ , Israel) was introduced. It non-invasively measures heart rate, heart rate variability, skin resistance, resistance variability, temperature and movement to calculate a nociception level (NoL) index. The NoL index range starts at zero, which is equivalent to being painless, and goes up to a value of 100. The validity and reliability of NoL monitoring is the content of current studies.

**Objective:**

We tested the hypothesis if the use of the PMD200 significantly reduces opioid consumption during da Vinci prostatectomy.

**Material and methods:**

A total of 50 male patients were included in this randomized, single blinded study. Exclusion criteria were arrhythmia because the pain monitoring device requires a sinus rhythm for reliable results. Patients received a weight-adjusted sufentanil bolus (0.3 µg/kg ideal body weight) during induction of anesthesia. Additionally, they received 10 µg of sufentanil before skin incision. Both groups received total intravenous anesthesia with propofol and continuous muscle relaxation through cis-atracurium.

In the control group (CONT; *n* = 26), a standardized sufentanil bolus of 10 µg were administered by common criteria (heart rate/blood pressure increase, lacrimation, gut feeling) at the anesthesiologistʼs discretion. In the intervention group (INT; *n* = 24), patients received the standardized sufentanil bolus when the NoL index was above 25 for 2 min, which corresponds to the manufacturerʼs recommendation. The NoL index and bolus administrations were recorded for every patient. In the control group, the display of the pain monitor showing the NoL index was not visible for the anesthesiologist. Postoperatively, pain/nausea scores and piritramide consumption were taken every 10 min for 1h in the recovery room. None of the patients had prior chronic pain with long-term use of painkillers.

Statistics were done using Mann-Whitney U‑test, Kolmogorov-Smirnov test and Levene test.

**Results:**

Sufentanil bolus administrations, normalized for duration of surgery, were not significantly lower in the intervention group (*p* = 0.065). We noticed a significant difference in variation of opioid administrations (*p* = 0.033). Sufentanil boluses per hour in the INT were normally distributed (*p* = 0.2), whereas in CONT they were not (*p* = 0.003). Postoperative data like nausea, opioid consumption and pain scale showed no differences between groups.

**Conclusion:**

The use of PMD200 did not significantly reduce cumulative opioid consumption. Following on we must reject the initial hypothesis. The difference in sufentanil bolus variances may point to an individualized antinociceptive therapy when NoL monitoring is used. We suppose patients with high opioid demands are detected and patients with low opioid demands did not receive unnecessary opioids. This assumption is only true if the PMD200 measures the entity pain. Further studies with more participants during surgery with higher tissue damage could lead to more convincing data and conclusions.

## Hinführung

Für eine patientenorientierte Narkose spielen objektive Messparameter eine entscheidende Rolle. Messungen von Hypnosetiefe und Muskelrelaxierung sind im Gegensatz zur Schmerzmessung seit Jahren etabliert. Seit Kurzem ist ein neuer Monitor zur Schmerzmessung verfügbar (PMD200 (Pain Monitoring Device; Fa. Medasense Biometrics™ Ltd., Ramat-Gan, Israel)) [2]. Aktuelle Studien weisen auf eine Reduzierung der intraoperativen Schmerzmittelgabe und des postoperativen Schmerzes hin [9, 10]. Daher wurde im folgenden die Hypothese überprüft, ob die Verwendung des PMD200 den Bedarf von Analgetika während einer Da-Vinci-Prostatektomie (roboter-assistiertes Chirurgiesystem; Intuitive Surgical Inc., Sunnyvale, CA, USA) reduziert.

## Methode

### Ethik und Patienten

Die Studie wurde durch die Ethikkommission der Medizinischen Fakultät am Universitätsklinikum Carl Gustav Carus Dresden genehmigt (BO-EK-32407020). Eine Registrierung erfolgte im Deutschen Register Klinischer Studien (DRKS00023232). Die schriftliche Einwilligung in die Teilnahme wurde nach den chirurgisch- und anästhesiologischen Aufklärungen eingeholt. Die Studie wurde einfach verblindet, prospektiv und randomisiert durchgeführt.

Eingeschlossen wurden geschäftsfähige, volljährige Patienten, die sich einer radikalen Prostatektomie mittels Da-Vinci-Roboter in Allgemeinanästhesie unterzogen. Ausschlusskriterium waren Herzrhythmusstörungen, da der Algorithmus des verwendeten Schmerzmonitors nur bei einem Sinusrhythmus verlässliche Daten generiert. Bei keinem der Studienteilnehmer waren vorbestehende Schmerzen oder eine regelmäßige Schmerzmitteleinnahme bekannt.

### Randomisierung und Zuordnung

Da zur Hypothese, dass die Verwendung des PMD200 den Schmerzmittelbedarf senkt, zu diesem Zeitpunkt, keine Voruntersuchungen vorlagen, wurden zur statistischen Fallzahlplanung 20 Anästhesieprotokolle bei Da-Vinci-Prostatektomien retrospektiv ausgewertet. Vor Einführung des Schmerzmonitors erhielten die Patienten pro Stunde Operationszeit im Mittel 0,7 ± 0,5 Injektionen von einem 10 µg Bolus Sufentanil. Unter Verwendung des Monitors betrug der Mittelwert 0,3 ± 0,4 Injektionen/h. Bei einem α von 5 % und einer Power von 90 % betrug die erforderliche Mindestgruppengröße 21. Die Poweranalyse wurde mit der Annahme durchgeführt, dass die Anwendung von NoL zu einer Reduktion der Sufentanilboligaben um 50 % führt. Aufgrund eines zu erwartenden Drop-outs wurde mit einer Gruppengröße von 25 geplant.

Insgesamt wurden 50 Patienten in die Studie eingeschlossen und mittels eines webbasierten Tools (https://www.randomizer.org/) randomisiert. Dabei wurden 24 Patienten der Interventionsgruppe (INT) und 26 Patienten der Kontrollgruppe (CONT) zufällig zugeordnet.

### Ablauf

Nach Anlage eines peripheren i.v.-Zugangs und Etablierung des Basismonitorings erfolgte die i.v.-Induktion der Allgemeinanästhesie mit 0,3 µg/kg (ideales Körpergewicht) Sufentanil (Fa. Hameln Pharmaceuticals GmbH, Hameln, Deutschland) und Propofol (Fa. Fresenius, Bad Homburg, Deutschland) nach klinischer Wirkung. Die Hypnose wurde durch eine kontinuierliche Gabe von Propofol aufrechterhalten. Die Steuerung der Narkosetiefe erfolgte mittels Neuromonitoring (Bispektralindex (BIS™); Fa. Medtronic GmbH, Meerbusch, Deutschland) mit einem Zielwert von 50. Die Patienten wurden kontinuierlich mit dem Muskelrelaxans Cisatracurium (Fa. Hikma Farmacêutica, Terrugem, Portugal) relaxiert. Das intraoperative Monitoring erfolgte gemäß dem klinikinternen Standard bei dieser Operation (5-Kanal-EKG, Pulsoxymetrie, invasive Blutdruckmessung, quantitative Relaxometrie).

**Figure 1:**
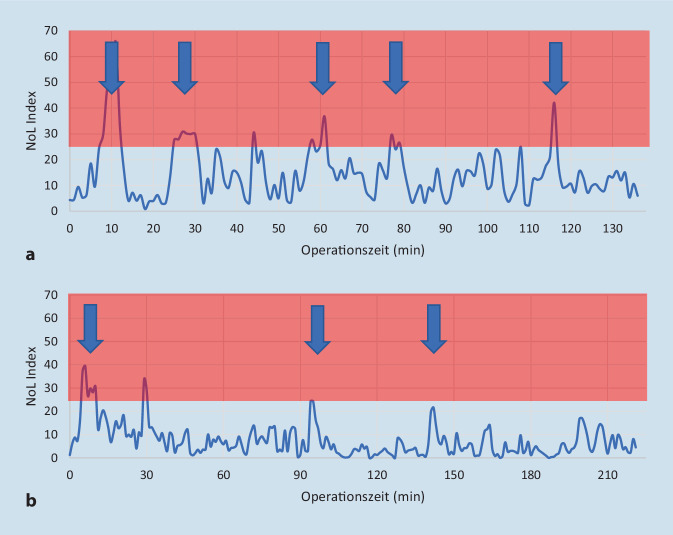

Bei 48 Patienten wurde, durch das PMD200, der einheitslose Schmerzindex NoL (nociceptor level) von Operationsbeginn bis -ende aufgezeichnet. Als Input-Variablen werden über einen Fingersensor nichtinvasiv Herzfrequenz, Herzfrequenzvariabilität, Hautwiderstand, Hautwiderstandsänderungen, Temperatur und Bewegung erfasst. Der NoL-Index kann Werte zwischen 0 und 100 annehmen [5]. Dabei entspricht der Wert 100 maximalem Schmerz und der Wert 0 keinem Schmerz. In der Kontrollgruppe (CONT) wurde der Monitor so platziert und abgedeckt, dass der NoL-Index intraoperativ nicht eingesehen werden konnte. Unmittelbar vor dem Hautschnitt erhielten alle Patienten 10 µg Sufentanil i.v. Weitere Schmerzmittelgaben erfolgten entweder durch Entscheidung des/der Anästhesisten/Anästhesistin nach üblichen Kriterien (Herzfrequenz‑/Blutdruckanstieg, Tränenfluss oder Bauchgefühl) (Gruppe CONT) oder aufgrund eines NoL-Index von mindestens 25 über 2 min (Gruppe INT) jeweils mit einem festgelegten 10 µg Sufentanilbolus. Ein NoL-Index von 10–25 ist laut Hersteller der angestrebte Zielbereich und wurde auch in anderen Studien verwendet [6, 9, 10]. Nach einem Sufentanilbolus wurden mindestens 5 min Abstand bis zur nächsten NoL-Index-Evaluation eingehalten. Abb. 1a zeigt stellvertretend für die INT-Gruppe eine NoL-Index-basierte Sufentanilboliapplikation; im Vergleich dazu die Daten eines Probanden der Gruppe CONT in Abb. 1b. In der Kontrollgruppe wurden bewusst keine Vorgaben zur Sufentanilgabe gemacht, um das Verfahren mit dem klinischen Alltag vergleichen zu können. Die Patienten erhielten während der Datenerfassung keine Nichtopioidanalgetika.

Nach Beendigung der Operation und Anästhesie wurden die Patienten mindestens 1 h im Aufwachraum nachbetreut. Dort erfolgten eine Erfassung des subjektiven Schmerzempfindens der Patienten (numerische Analogskala [NAS] von 0: keine bis 10: stärkste Schmerzen) und die Erfassung der Opioidnebenwirkung Übelkeit (NAS: 0: keine Übelkeit bis 10 = Erbrechen) im 10 min Abstand. Bei einem NAS-Wert ≥ 4 erhielten die Patienten 3 mg Piritramid (Fa. Hameln Pharmaceuticals GmbH, Hameln, Deutschland) i.v.

### Statistik

Die statistische Auswertung erfolgte bei nichtparametrischen und unabhängigen Variablen mittels Mann-Whitney-U-Test. Die Überprüfung auf Varianzhomogenität wurde mit der Levene-Statistik durchgeführt. Die Testung auf Normalverteilung erfolgte mit dem Kolmogorow-Smirnow-Test (H_0_: Daten sind normal verteilt; H_1_: Daten sind nicht normal verteilt). Der Test auf Homogenität der randomisierten Gruppen erfolgte mittels zweiseitigem t‑Test. *p* < 0,05 wurde als Signifikanzniveau angenommen.

## Ergebnisse

**Figure 2:**
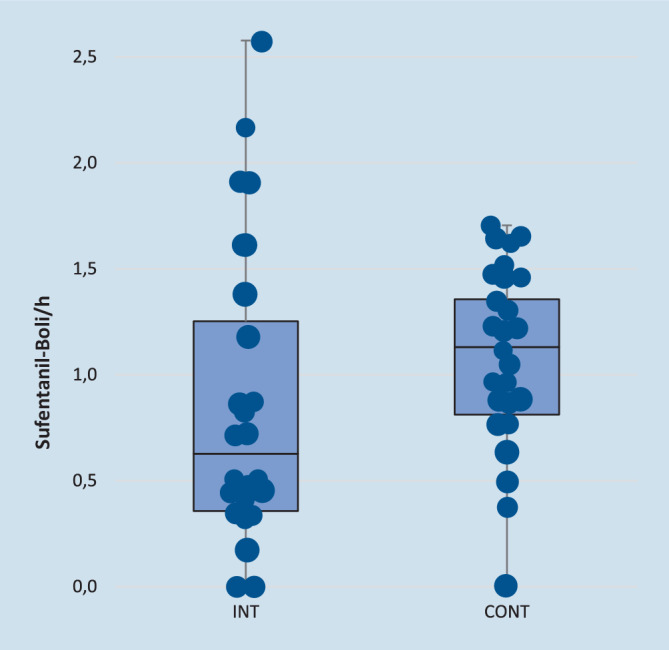

Alle 50 randomisierten Patienten haben an der Untersuchung teilgenommen. Bei 2 Patienten der CONT-Gruppe konnten aufgrund fehlerhafter Anbringung des Fingersensors und unterbrochener Stromzufuhr des PMD200 keine NoL-Werte erfasst werden. Die beiden Gruppen wiesen keine demografischen Unterschiede auf (Tab. 1). Die Anzahl der Sufentanilgaben wurde auf die Operationszeit normalisiert. Die Ergebnisse sind in den Tab. 2 und 3 zusammengefasst und in Abb. 2 dargestellt.

**Table 1:** 

Parameter	INT	CONT	*p*-Wert
Alter (Jahre) ± SD	64 ± 5	65 ± 5	n. s.
Größe (cm) ± SD	179 ± 6	178 ± 6	n. s.
Gewicht (kg) ± SD	81 ± 12	88 ± 15	n. s.
ASA (I/II/III)	5/16/3	2/20/4	n. s.

*INT* Interventionsgruppe, *CONT* Kontrollgruppe*, ASA* American-Society-of-Anesthesiologists-Klassifikation

**Table 2:** 

Parameter	INT	CONT	*p*-Wert
Anästhesie-Zeit (min) ± SD	260 ± 49	283 ± 53	0,13
Operationszeit (min) ± SD	181 ± 45	202 ± 53	0,15
Sufentanil, gesamt (µg) ± SD	60 ± 20	69 ± 16	0,07
Aufwachzeit (min) ± SD	5 ± 6	6 ± 6	0,35

*INT* Interventionsgruppe, *CONT* Kontrollgruppe*, Aufwachzeit* Operationsende bis zur Extubation

**Table 3:** 

Parameter	INT	CONT	Mann-Whitney-U-Test
MW ± SD	0,9 ± 0,7	1,1 ± 0,4	*p* = 0,065
Min/Max/Median	0/2,6/0,6	0/1,7/1,1	–
Quartil 25 %/75 %	0,4/1,3	0,8/1,4	–

*INT* Interventionsgruppe, *CONT* Kontrollgruppe

Patienten der INT-Gruppe erhielten im Mittel etwas seltener Sufentanilboli als Patienten der CONT-Gruppe. Der Unterschied ist weder statistisch signifikant noch, bei Betrachtung der Werte, klinisch relevant. Die Ausgangshypothese wurde daher verworfen.

Bei der Betrachtung der Verteilung der Häufigkeiten fanden sich Unterschiede zwischen den Gruppen (Abb. 2). Der Test auf Varianzhomogenität zeigte einen signifikanten Unterschied zwischen den Gruppen (*p* = 0,033). Die Verteilung entsprach in der CONT-Gruppe (*p* = 0,20) im Gegensatz zur INT-Gruppe (*p* = 0,003) statistisch einer Normalverteilung.

Zusätzlich untersuchten wir, ob zu den Zeitpunkten, an denen Sufentanilboli gegeben wurden, der NoL-Index ein Schmerzempfinden bestätigte. In der CONT-Gruppe wurden insgesamt 69 und in der INT-Gruppe 57 Sufentanilboli verabreicht. Der gleichzeitig erfasste NoL-Index zeigte, dass in der CONT-Gruppe in 27 % und in der INT-Gruppe in 91 % der Fälle die Gabe laut Protokoll gerechtfertigt gewesen war (Tab. 4). Dieser Unterschied war statistisch signifikant (χ^2^-Test: *p* < 0,000005).

**Table 4:** 

Gruppe	Anzahl von Sufentanilboli	Dabei NoL > 25 (2 min) ≙ Schmerz
CONT	69	18
INT	57	52

In der INT-Gruppe kam es zu 5 Protokollverletzungen, es wurde 5‑mal kein erhöhter NoL-Index vor einer Sufentanilapplikation aufgezeichnet

*INT* Interventionsgruppe, *CONT* Kontrollgruppe

7 min nach der Bolusapplikation (Wirkmaximum von Sufentanil) war in 46 von 52 Fällen der INT-Gruppe eine deutliche NoL-Index-Erniedrigung unter 25 und über 2 min messbar. Somit war laut NoL-Index in 9 von 10 Fällen der Bolus ausreichend. In den 6 Fällen, wo dies nicht der Fall war, sorgte ein erneuter Bolus für eine ausreichende NoL-Index-Senkung. Des Weiteren wurden in der CONT-Gruppe 65 aus insgesamt 83 Sufentanilboli appliziert ohne, dass eine passende NoL-Index-Erhöhung über 25 messbar war.

Im Aufwachraum zeigten sich keine signifikanten Unterschiede im mittleren postoperativen Schmerz (*p* = 0,55) oder in der postoperativen Piritramidgabe (*p* = 0,89). Über Übelkeit klagte lediglich einer von 50 Patienten.

## Diskussion

Die Ausgangshypothese, dass die Verwendung des NoL-Monitors die Schmerzmittelgabe bei Da-Vinci-Prostatektomien signifikant reduziert, wurde verworfen. Weitere Studien bestätigen unsere Ergebnisse [6, 9]. Bisher konnte nur in einer Studie eine signifikante Reduktion der intraoperativen Opioidgabe gezeigt werden [10].

Nach genauer Betrachtung der statistischen Analysen zeigten sich deutliche Unterschiede im Gruppenvergleich: In der INT-Gruppe erhielten sowohl mehr Patienten weniger Boli, aber auch einige deutlich mehr Boli als in der CONT-Gruppe (Abb. 2). Eine mögliche Interpretation der unterschiedlichen Varianz der verabreichten Sufentanilboli ist, dass die Schmerzmittelgabe in der INT-Gruppe individualisierter erfolgte, d. h., es wurden nichterforderliche Schmerzmittelgaben vermieden, und gleichzeitig detektierte die NoL-Messung einzelne Patienten mit deutlich erhöhtem Schmerzmittelbedarf. Diese Schlussfolgerung ist allerdings nur unter der Voraussetzung zulässig, dass das PMD200 auch tatsächlich die Entität Schmerz misst. Dass NoL-Monitore Schmerzstimuli mit größerer Sensitivität und Spezifität erkennen als andere singuläre Parameter wie Herzfrequenz oder Blutdruck, wurde bereits gezeigt [5, 8, 14]. Auch ein direkter Zusammenhang zwischen einer Opioidgabe und folgender Senkung des NoL-Index wurde nachgewiesen [12].

Welche Einflüsse auf das NoL-Monitoring während einer Allgemeinanästhesie zu berücksichtigen sind, unterliegt weiteren Untersuchungen [4]. Vasopressoren scheinen den NoL-Index nur in sehr geringem und klinisch irrelevantem Maße zu beeinflussen [11]. Auch β_1_-Rezeptoren-Blocker zeigten in einer Untersuchung keinen relevanten Einfluss auf den NoL-Index [3].

Hinweise, dass eine NoL-Index-basierte Opioidapplikation den postoperativen Schmerz signifikant reduziert, konnte unsere Studie nicht bestätigen [9]. Grund dafür könnte das generell niedrige postoperative Schmerzniveau nach Da-Vinci-Prostatektomien sein. Darüber hinaus fällt es vielen Patienten schwer, in den ersten Stunden nach der Prostatektomie zwischen Wundschmerz und Blasendruck zu differenzieren.

In den letzten Jahren erhielten weitere Schmerzmonitore, mit unterschiedlichen Messverfahren, die Zulassung. Neben dem Multiparameter-messenden PMD200 besteht auch die Möglichkeit, den Surgical Pleth Index (SPI; Fa. GE Healthcare, Helsinki, Finnland) zu ermitteln. Der SPI bestimmt mithilfe eines Algorithmus aus der Pulsplethysmographie ein Schmerzlevel und verzichtet dabei auf das Bestimmen mehrerer Parameter [13]. Ein weiteres Messverfahren, welches nur einen Parameter erfasst, stellt der Analgesia Nociception Index (ANI; MDoloris Medical Systems, Loos, Frankreich) dar, welcher durch Herzfrequenzvariabilität ein Schmerzlevel berechnet [7]. Der Cardiovascular-Depth-of-Analgesia(CARDEAN)-Monitor (Alpha‑2 Ltd, Lyon, Frankreich) bestimmt einen Schmerzindex unter Berücksichtigung von Blutdruck und Herzfrequenzmessung [15]. Auch der Pupillary Pain Index (PPI, z. B. Algiscan, ID Med, Marseille, Frankreich) ist Bestandteil aktueller Untersuchungen. Das Pupillometer zeichnet dabei Pupillenweite und Pupillenlichtreflex auf, um das Schmerzlevel zu bestimmen [1, 6]. Der Stellenwert dieses Geräts liegt aber wahrscheinlich eher im postoperativen Setting.

Neue Studiendesigns zielen auf einen Vergleich der Schmerzmonitore ab. Dabei konnte bisher kein Verfahren als eindeutig überlegen definiert werden. Zwar unterscheiden sich die intraoperativen Opioidgaben teilweise signifikant, jedoch ging eine geringere Gesamtmenge an Opioid mit einem erhöhten Stresshormonlevel einher [6].

In der Zusammenschau mit den Ergebnissen unserer Studie ist das Einsparen von Opioiden durch die verschiedenen Schmerzmonitore im Mittelwert klinisch nicht bedeutsam. Dazu kongruent ist in unseren postoperativen Daten ein relevanter Effekt durch den Schmerzmonitor nicht darstellbar. Hingegen soll das Ziel sein, eine individualisierte Schmerztherapie für die Patient*Innen zu gewährleisten.

## Limitationen

Bei 2 INT- (5-mal Sufentanilgabe ohne erhöhten NoL-Index) und 3 CONT-Patienten (inkorrekte Sufentanileinleitungsdosis) kam es zu Verstößen gegen das Studienprotokoll. Diese Patienten sind Teil der Datenanalyse („Intention-to-treat“-Analyse).

Es handelt sich bei der Da-Vinci-Prostatektomie um eine Operation mit geringem Schmerzreiz. Für zukünftige Untersuchungen wären Operationen mit größeren Wundflächen und somit höherem Opioidbedarf interessant. Mit 55 bis 75 Jahren handelte es sich weiter um eine sehr eng gefasste Altersgruppe an Männern, und daher sind die Ergebnisse nicht ohne Weiteres auf die Allgemeinbevölkerung übertragbar.

## Fazit für die Praxis

Das NoL-Monitoring hat während einer Da-Vinci-Prostatektomie keinen signifikanten Einfluss auf die Gesamtzahl der verabreichten Sufentanilboli. Die unterschiedliche Verteilung der Sufentanilboli in den 2 Gruppen interpretieren wir als möglichen Hinweis, dass mit dem PMD200 Patienten mit erhöhtem Schmerzmittelbedarf detektiert werden können und nichterforderliche Schmerzmittelgaben bei anderen Patienten vermieden werden. Dadurch wird eine individualisierte Schmerztherapie ermöglicht, und Patienten mit erhöhten oder sehr geringen Opioidbedarf werden identifiziert. Grundvoraussetzung für diese Hypothese ist, dass der PMD200 die Entität Schmerz erfasst. Weitere Untersuchungen bei Operationen mit größerem Schmerzreiz könnten hier Klarheit bringen.
